# Circulating Anti-Müllerian hormone in a cohort-study of women with severe obesity with and without polycystic ovary syndrome and the effect of a one-year weight loss intervention

**DOI:** 10.1186/s12958-022-01022-0

**Published:** 2022-10-29

**Authors:** Josefin Kataoka, Ingrid Larsson, Eva Lindgren, Li Oskarson Kindstrand, Johanna Schmidt, Elisabet Stener-Victorin

**Affiliations:** 1 Institute of Neuroscience and Physiology, Department of Physiology, Sahlgrenska Academy, University of Gothenburg, Box 430, 405 30 Gothenburg, Sweden; 2 Department of Obstetrics and Gynaecology, Sahlgrenska University Hospital, Gothenburg, Sweden; 3 Department of Gastroenterology and Hepatology, Sahlgrenska University Hospital, Gothenburg, Sweden; 4 Institute of Medicine, Sahlgrenska Academy, University of Gothenburg, Box 428, 405 30 Gothenburg, Sweden; 5 Institute of Clinical Sciences, Department of Obstetrics and Gynaecology, Sahlgrenska Academy, University of Gothenburg, 416 85 Gothenburg, Sweden; 6 Department of Physiology and Pharmacology, Karolinska Institutet, Biomedicum, B5, 171 77 Stockholm, Sweden

**Keywords:** Polycystic ovary syndrome, Anti-Müllerian hormone, Severe obesity, Weight loss

## Abstract

**Background:**

Women with polycystic ovary syndrome (PCOS) have high circulating anti-Müllerian hormone (AMH) levels which is correlated with antral follicle count and polycystic ovarian morphology and negatively correlated with body mass index (BMI). Moreover, diet-induced weight loss in women with PCOS and overweight or obesity, reduce or normalize AMH-levels. There is, however, no previous study investigating the circulating AMH levels in women with severe obesity and how a structured diet-induced weight loss program affects circulating AMH levels in these women. Therefore, this study aims to investigate circulating AMH levels in a population of women with severe obesity (BMI ≥ 35 kg/m^2^) with and without PCOS, as diagnosed by the NIH-criteria, and to investigate the effect of a one-year weight loss program with a very low-energy diet (VLED) on circulating levels of AMH.

**Methods:**

In a prospective cohort-study, were 246 women with severe obesity were screened for PCOS diagnosis with the NIH-criteria, circulating AMH and anthropometry were measured at baseline and after a 12-month weight loss intervention with very low-energy diet (VLED).

**Results:**

Mean BMI was 39.9 ± 4.7 (PCOS), 39.6 ± 4.3 (non-PCOS) *P* = 0.960. Circulating AMH was higher in women with PCOS (5.47 ± 4.89 µg/L) compared with non-PCOS (2.66 ± 3.71 µg/L) *P* < 0.001 and was positively correlated with circulating total testosterone in both groups. Next, we performed ROC-analyses, and show that circulating AMH could not discriminate women with PCOS and severe obesity from non-PCOS women with severe obesity. Finally, a one-year weight reduction program does not affect circulating AMH levels despite significant weight loss neither in women with PCOS, nor without PCOS and severe obesity.

**Conclusion:**

Women with severe obesity and PCOS have elevated levels of circulating AMH compared to women without the syndrome. AMH-levels could not discriminate women with PCOS from non-PCOS because of low sensitivity and specificity. Significant weight loss was not associated with changes in circulating AMH levels, neither in women with, nor without PCOS and severe obesity. These results imply that in women with severe obesity, a greater weight loss may be needed to improve reproductive features, independent of PCOS diagnosis.

**Trial registration number:**

Clinical trial.gov: NCT01319162.

**Supplementary Information:**

The online version contains supplementary material available at 10.1186/s12958-022-01022-0.

## Background

Polycystic ovary syndrome (PCOS) is an endocrine disorder which affects approximately 10% of women of reproductive age [1, 2]. It is characterized by hyperandrogenism and anovulatory infertility, and strongly linked to insulin resistance with an increased risk of developing type 2-diabetes and cardiovascular disease [3–5]. Moreover, obesity aggravates all symptoms. Depending on diagnostic criteria, the prevalence of PCOS varies, and we have recently reported a prevalence of 25.6% in women with severe obesity, using the National Institutes of Health (NIH)-criteria [6].

Anti-Müllerian hormone (AMH) is a glycoprotein produced in the granulosa-cells of pre-antral and antral follicles and is important in follicular development by preventing follicular growth. Women with PCOS have an accumulation of small antral follicles resulting in a higher number of antral follicles and an excessive production of AMH, leading to high levels of circulating AMH [7–9]. AMH acts on the ovary by inhibiting FSH-induced aromatase activity, which contributes to hyperandrogenism, and by inhibiting antral follicle growth contributing to the polycystic ovarian morphology (PCOM). Recent findings demonstrate that elevated circulating AMH levels increase the secretion of gonadotropin releasing hormone (GnRH) which increases secretion of luteinizing hormone (LH) [10], leading to increased androgen production from the ovaries. Thus, AMH could exacerbate ovarian hyperandrogenism in women with PCOS [10]. In line with these findings, studies have shown that in women with PCOS, circulating AMH is correlated with a more severe PCOS-phenotype [11, 12]. AMH is strongly correlated to antral follicle count (AFC) [13], and there is a correlation between AMH and polycystic ovarian morphology (PCOM), however current evidence does not support its use as a surrogate marker for PCOM since there are no standardized assays or established cut-off levels [14].

Existing studies show contradictory results regarding the correlation between AMH and BMI.

Some studies have shown that AMH-levels are negatively correlated with BMI in women with [15], and without PCOS, which suggests that excess adiposity might compromise the ovarian reserve [16–18]. Other studies show no association between AMH and BMI in women with PCOS with normal and overweight [19, 20], or in women without PCOS and normal to obesity class 2 [21–23].

There is also evidence of reduced or normalized AMH-levels after diet-induced weight loss in women with PCOS and overweight or obesity [24–26], whereas other studies show improved reproductive function without concomitant changes in AMH- levels [27, 28]. However, to our knowledge, there is no study to date investigating AMH-levels in women with PCOS and severe obesity, compared to controls, and the effect of diet-induced weight loss on circulating AMH.

The aim of this study was to investigate circulating AMH levels in a population of women with severe obesity (BMI ≥ 35 kg/m^2^) with and without PCOS, as diagnosed by the NIH-criteria. Secondly, we aim to investigate the effect of a one-year weight loss program with a very low-energy diet (VLED) on circulating levels of AMH.

## Methods

### Design setting and participants

This study was conducted from 2011 to 2016 and included 298 women who were referred for weight loss treatment to the Regional Obesity Center at Sahlgrenska University hospital in Gothenburg, Sweden. To be eligible, women needed to be between 18 and 50 years of age, with no reported signs of menopause. After verbal and written consent, the participants were screened for the diagnosis of PCOS according to the NIH-criteria (presence of clinical and/or biochemical hyperandrogenism (with cut-off levels of modified Ferriman Gallwey (mFG) score ≥ 6, or free testosterone (fT) > 0.035 nmol/L, or total testosterone > 1.2 nmol/L or free androgen Index (FAI) > 5), and oligo-/anovulation and exclusion of other causes) and has been described in detail earlier [6].

Women who were pregnant or breastfeeding within the last six months, or had reported signs of climacteric symptoms, or a language barrier or reduced ability to understand information were excluded.

### Measurements

At baseline, participants completed a questionnaire regarding PCOS, including information on menstrual cycles, self-reported modified Ferriman-Gallwey (mFG)-score and previous diagnosis of PCOS. Body weight was measured to the nearest 0.1 kg on calibrated scales with the subject dressed in indoor clothes and no shoes. Height was measured to the nearest 0.5 cm using a wall mounted stadiometer. BMI was calculated by dividing weight by squared height (kg/m^2^). Fasting blood samples were collected for analyses of total testosterone, sex hormone binding globulin (SHBG) and AMH. Follow-up at 12 months followed the same procedure. Blood samples (except AMH) were analysed at the ISO-accredited laboratory (ISO 15189:2012, ISO 22870:2016) at Sahlgrenska University Hospital, Gothenburg, Sweden. Testosterone was measured with electrochemiluminiscent immunoassay with competitive analysis (ECLIA) (COBAS 8000 Roche Diagnostics Scandinavia AB, Sweden). The CV was 6% at 2.0 nmol/L. The lower detection limit was 0.4 nmol/L. Free testosterone was calculated by using total testosterone and SHBG and assuming a fixed albumin concentration of 43 g/L, [29]. Free androgen index (FAI) was calculated using total testosterone divided by SHBG x100.

Serum concentration of anti-Müllerian hormone (AMH) was measured at the institute of Physiology and Pharmacology at Karolinska Institutet Stockholm Sweden, using the Ultra-Sensitive AMH/MIS ELISA (AL-105, Ansh Labs, Texas, USA). The AMH ELISA is a three-step sandwich-type Immunoassay, where the samples are added to an AMH antibody coated plate. The assay uses stabilized recombinant human AMH as calibrators, with an analytical measure range of 0.08 to 14.2 ng/ml.

### Intervention

All participants started a 12-month dietary intervention with an initial 12-week period of a very low energy diet (VLED), followed by a reintroduction of solid food according to a prescheduled plan. During the 12-month treatment period, each participant followed a plan with monthly visits to study dieticians, with support and counselling of an energy-restricted diet. At the 12 months follow-up, baseline assessments were repeated. Detailed description of the intervention has been published previously [6].

### Statistical analysis

Sample size was based on estimation of prevalence of PCOS among women with severe obesity and has previously been published [6]. Statistical analyses were performed using IBM SPSS statistics version 25.0 software. Results are presented as mean ± SD. Comparisons between groups of women with and without PCOS at baseline were determined by Mann-Whitney U test and *P-* values adjusted for age by ANCOVA. Changes from baseline to 12-month were analysed by Wilcoxon signed ranks test. *P* < 0.05 was considered statistically significant. ROC-analyses and area under the curve was used to calculate sensitivity and specificity of serum concentration of AMH and other endocrine parameters to discriminate women with PCOS from women without PCOS.

## Results

**Fig. 1:**
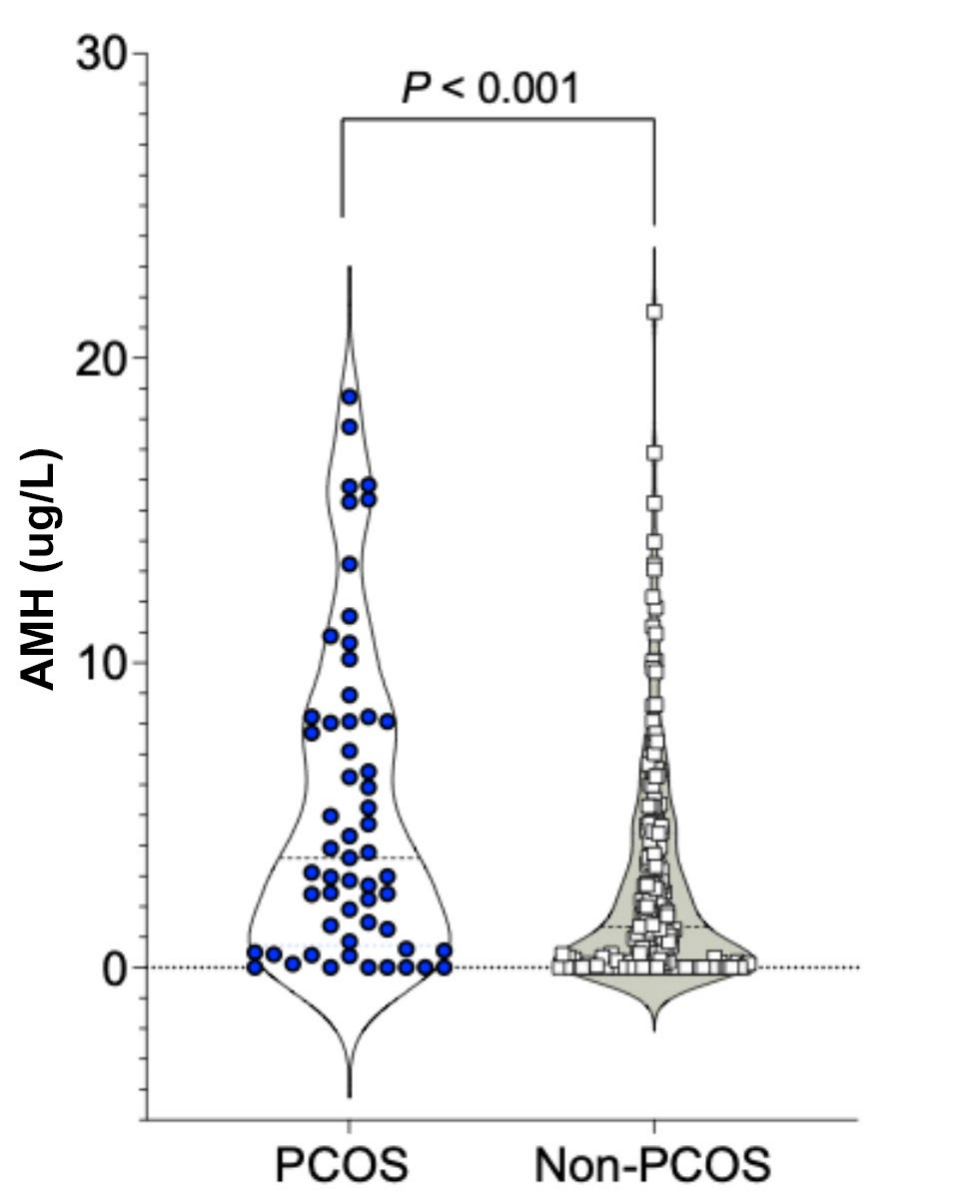
Circulating levels of AMH in women with severe obesity with PCOS (n = 63) and without PCOS (n = 183) at baseline

In total 298 women accepted to join the study, but complete data to definitively diagnose PCOS was not available for 52 participants, and thus they were excluded from analyses. This left 246 women with severe obesity to be included in the study, and screened for PCOS diagnosis according to the NIH-criteria (PCOS n = 63; non-PCOS n = 183). Age ranged from 18 to 50 years in both women with and without PCOS. Anthropometric and hormonal parameters at baseline are shown in Supplemental Tables 1, as published previously [6]. The serum levels of AMH were higher in women with PCOS (5.47 ± 4.89 µg/L), than in women without PCOS (2.66 ± 3.71 µg/L) (*P* < 0.001, adjusted for age) (Fig. 1, Supplemental Table 1). The diagnosis of PCOS was set with the NIH-criteria, which requires menstrual disturbances and hyperandrogenemia. In total 62 women with PCOS presented with oligo-/amenorrhea and 1 woman had stated a previous diagnosis of PCOS. In women without PCOS, 29 women presented with oligo-/amenorrhea, 151 women with regular menses, and 3 women did not report menstrual status but did not have any hyperandrogenemia, and thus no PCOS-diagnosis.

Moreover, AMH had a positive correlation with total testosterone but not with SHBG and BMI (Table 1).

**Table 1:** Correlation between baseline values of AMH, total testosterone, free testosterone, SHBG and BMI.

	All n = 246	PCOS n = 63	non-PCOS n = 183
	AMH	AMH	AMH
T (nmol/L)	0.493	0.275*	0.268*
f-T (nmol/L)	0.543	0.202	0.319*
SHBG (nmol/L)	0.029	0.076	0.058
BMI	-0.429	0.004	-0.025

PCOS polycystic ovary syndrome; AMH, anti-Müllerian hormone; SHBG, sexual hormone binding globulin; T, testosterone; f-T, free testosterone; BMI, body mass index

Correlation analyses were made with Spearmans test, * *P* < 0.05.

**Fig. 2:**
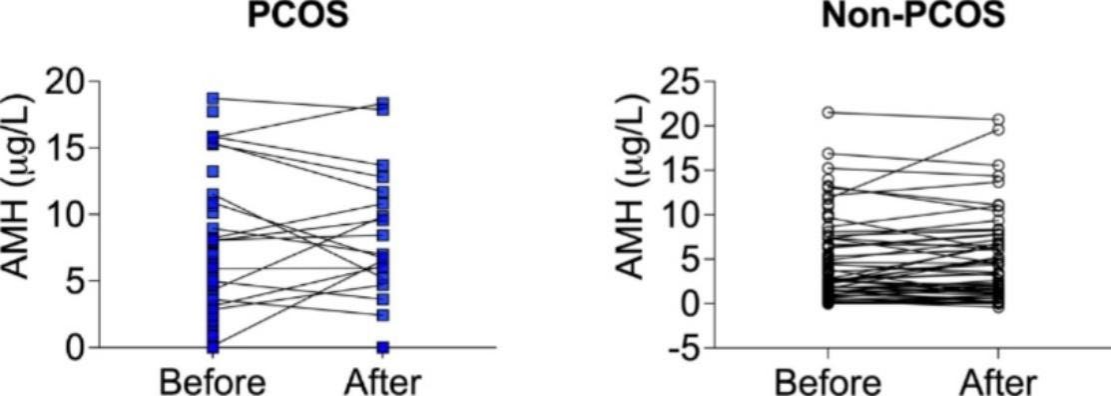
Circulating levels of AMH in women with severe obesity with PCOS (n = 19) and without PCOS (n = 64) did not change from before to after intervention

In total, after 12 months dietary intervention, AMH-data was available for 83 women. Women that dropped out did not differ in baseline characteristics from those who completed the intervention. After the intervention, a significant weight loss was observed from baseline in both women with and without PCOS; non-PCOS 111.1±15.5 kg to 97.0±16.5 kg (p < 0.001) and PCOS 106.4±12.9 kg to 94.5±12.3 kg (p > 0.001), respectively. AMH did not change in either of the two groups (Fig. 2).

**Fig. 3:**
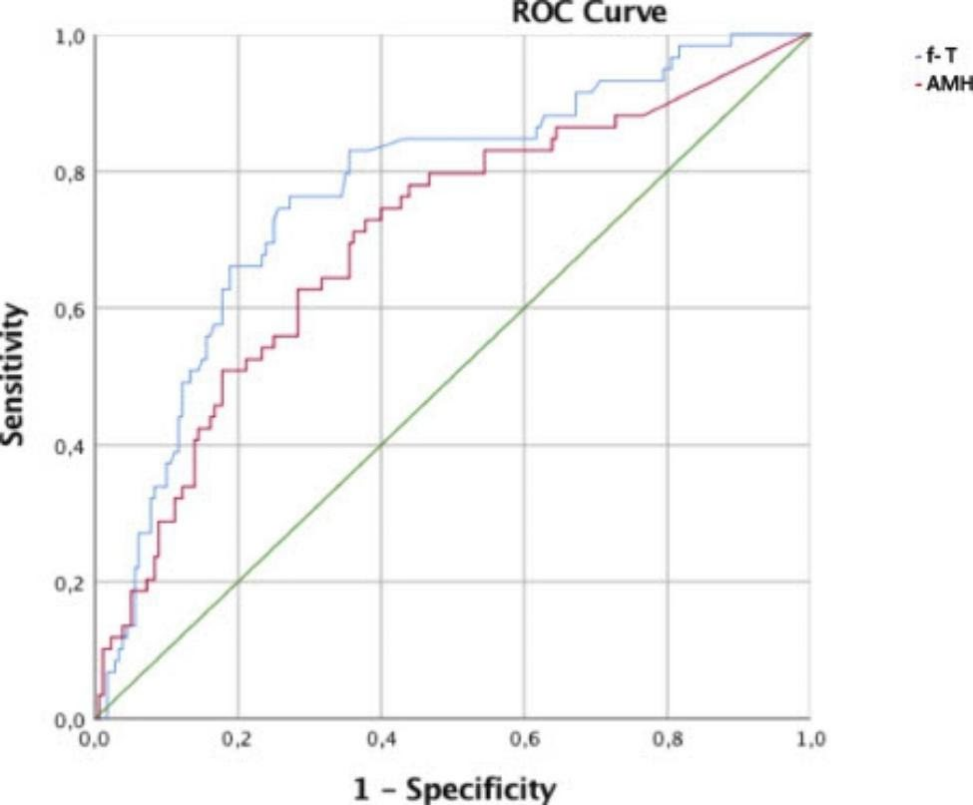
ROC curves for the detection of PCOS using circulating levels of free Testosterone (f-T) and AMH (anti-müllerian hormone)

When using ROC analyses to explore the ability of serum concentrations of AMH and other endocrine parameters to distinguish women with PCOS from women without PCOS, the area under the curve (ROC_AUC_) was 0.770 (95% CI, 0.700–0.840) for fT and 0.701 (95% CI, 0.622–0.780) for AMH (Fig. 3). Because AMH had a low ROC_AUC_, a decision threshold with appropriate sensitivity and specificity could not be generated.

## Discussion

To our knowledge, this is the first study investigating the effects of weight loss on AMH in women with PCOS and severe obesity. Here we demonstrate that women with PCOS and severe obesity have higher circulating AMH levels than women with similar BMI without PCOS. Our findings support previous observations in women with PCOS both with normal weight and with obesity [7–9, 11]. Moreover, we found no effect of significant weight loss induced by a 12-month weight loss program on circulating AMH, in women with or without PCOS.

One of the main characteristics of PCOS is the large number of pre-antral and antral follicles, and it is the granulosa cell layer of these follicles that produce AMH [13, 30, 31]. Women with PCOS, independent of BMI, have a 2–4 fold higher circulating AMH [7], which likely reflects the increased number of antral follicles and has been proposed to be a surrogate marker for AFC and PCOM [32]. The recently published evidence based international guidelines for PCOS do not support circulating AMH as a diagnostic marker and do not recommend AMH to replace PCOM measured by ultrasound [14].

The elevated circulating AMH has also been shown to be positively associated with circulating testosterone, and studies have shown that AMH reflects the severity of PCOS with the correlation between phenotypic presentation and AMH levels [13, 33]. In line with these observations, the highest circulating AMH levels have been found in women with all three diagnostic criteria (hyperandrogenism, PCOM, and anovulation), both in women with normal weight and obesity [11]. In the present study, women were diagnosed according to the NIH criteria, i.e.hyperandrogenism, and anovulation, and indeed, AMH was correlated with circulating androgens. Previous studies have shown a negative association between AMH and BMI in women with overweight and obesity, which was not seen in this study. This might be due to the limited variation in BMI, not including women with overweight and obesity grade 1.

In ROC analyses, circulating free testosterone had higher sensitivity and specificity than AMH, thus AMH could not be used to discriminate between women with severe obesity with and without the syndrome with enough precision. This is in line with one previous study [12], but recently, another study showed that AMH could be a robust method for diagnosing PCOM, with high sensitivity and specificity [34]. Both studies were done on women with normal and overweight and did not include women with severe obesity. The high sensitivity and specificity observed in androgen measures in this study are in line with our previous observation in women with normal weight and women with obesity with PCOS [35].

More than 50% of women with PCOS have obesity, and obesity aggravates all PCOS symptoms [36]. Interventions leading to weight loss are considered the first line treatment for women with PCOS and obesity [37]. Weight loss improves all features of the disease, via decreased insulin resistance and lower androgen levels, leading to improvement in ovarian function and lower AMH [37]. Research on metformin therapy has shown that it lowers circulating AMH, due to improvements in insulin function and a decreased number of antral follicles, and lower androgen levels [38]. Weight loss also increases ovulation and decreases circulating AMH [24–26].

Previous weight loss studies on women with PCOS suggest that a weight loss of ≥ 5% has an overall improvement of symptoms [39]. However, studies include small sample sizes. Guidelines for the management of PCOS suggest ≥ 5% weight loss as a goal in clinical practice and not as evidence-based recommendations [14].

In this study, we found no effect on circulating AMH after significant weight loss, both in women with or without PCOS. AMH levels are also associated with age, with lower levels in women after menopause, and therefore, in women of reproductive age, a large decrease could not be expected due to age. Previously published data on this cohort showed that even though the participants lost 12% in weight, a large part of them still had severe obesity as well as hyperandrogenemia, and metabolic parameters were not improved [6].

In this group of women with severe obesity, a weight loss beyond 12% could be needed to detect improvements, both in androgen levels and in circulating AMH. Although there was a significant weight loss in this study (range, -44.9 kg to + 11.6 kg), after 12 months, included women had a mean BMI of 33.8 ±4.0 kg (PCOS) 35.1± 5.4 kg (non-PCOS) and were still classified as having obesity or severe obesity, which is associated with IR and ovulatory dysfunction, and possibly, therefore, we could not find decreased levels of circulating AMH. Lifestyle-induced weight loss in adolescent girls with PCOS, with a change in BMI of -3.8 ± 1,7 kg/m^2^ resulted in a decrease in AMH [24]. Moreover, in women with PCOS and a BMI over 45, bariatric surgery leading to a weight loss of 19% resulted in a decrease in AMH [40]. Both studies led to a change in BMI class from obesity or morbid obesity to overweight or normal weight.

The strength of this study was the relatively large study sample, all with severe obesity at baseline. Another strength was the well-defined PCOS population, with high mean values of fT and mFG-score. Limitations included the selection of patients from an obesity center and not from the general population, the relatively large age span (18 to 50 years), and the high number of drop-outs during the intervention part of the study. Although a high drop-out rate of up to 80% is common in weight loss studies [41, 42], follow-up data must be interpreted with caution. Of note is that women without PCOS were older than women with PCOS, and even though analyses were adjusted for age, AMH levels could be decreased in this group due to age. Moreover, the initial aim was to perform a vaginal ultrasound at baseline to include PCOM in the diagnosis. However, in addition to logistical problems, it was impossible to perform vaginal ultrasound with the adequate result on these women, due to the severity of obesity, even though the examination was performed by a highly experienced ultrasound specialist. Therefore, we had to stop this examination and diagnose the women according to the NIH criteria [6].

Taken together, in women with severe obesity, circulating AMH is higher in women with PCOS and positively correlated with androgen levels, but AMH did not decrease with significant weight loss in women with or without PCOS. These results imply that in women with severe obesity, a greater weight loss may be needed to improve reproductive features, independent of PCOS diagnosis.

## Electronic supplementary material

Below is the link to the electronic supplementary material.

Supplementary Material 1

## Data Availability

Data will be available on request.
