# Supplementary material for: Circulating Anti-Müllerian hormone in a cohort-study of women with severe obesity with and without polycystic ovary syndrome and the effect of a one-year weight loss intervention
Source: Reprod Biol Endocrinol. 2022 Oct 29;20:153. doi: 10.1186/s12958-022-01022-0 (PMC9617381; doi:10.1186/s12958-022-01022-0)
Supplement: Supplementary file 1 — Supplementary Material 1 [file 12958_2022_1022_MOESM1_ESM.docx]

**Supplemental table 1.** Baseline characteristics in women with severe obesity with and without PCOS. Previously published (6).

|  | PCOS  n =63 | Non-PCOS  n =183 | p_a_ | p_b_ |
| --- | --- | --- | --- | --- |
| Age | 33.0 ± 8.4 | 37.7 ± 8.7 | **<0.001** | - |
| AMH (μg/L) | 5.47 ± 4.89 | 2.66 ± 3.71 | **<0.001** | **0.001** |
| Weight (kg) | 111.0 ± 16.8 | 110.7 ± 14.6 | 0.861 | 0.938 |
| BMI | 39.9 ± 4.7 | 39.6 ± 4.3 | 0.787 | 0.960 |
| BMI-range | 32.6 - 50.1 | 30.4 - 58.7 |  |  |
| T (nmol/L) | 1.62 ± 0.57 | 1.15 ± 0.50 | **<0.001** | **<0.001** |
| SHBG (nmol/L) | 28.59 ± 20.33 | 39.45 ± 29.60 | **<0.001** | **0.042** |
| f-T (nmol/L) | 0.033 ± 0.014 | 0.021 ± 0.012 | **<0.001** | **<0.001** |
| FAI | 7.32 ± 3.93 | 3.97 ± 3.28 | **<0.001** | **<0.001** |
| mFG-score | 10.89 ± 7.52 | 4.57 ± 5.13 | **<0.001** | **<0.001** |

PCOS, polycystic ovary syndrome; AMH, anti-Müllerian hormone; BMI, body mass index; SHBG, sexual hormone binding globulin; FAI, free-androgen index; T, testosterone; f-T, free testosterone; mFG-score, modified Ferriman-Gallwey-score.

Values are presented as mean ± standard deviation (SD).

p_a_ was calculated according to Mann-Whitney-U test.

p_b_ is adjusted for age and calculated with ANCOVA.

p<0.05 was considered statistically significant.
